# Association between heat and air pollution (PM2.5 and black carbon) exposure in pregnancy and preterm birth in low- and middle-income countries: a systematic review and meta-analysis

**DOI:** 10.1136/bmjpo-2025-003428

**Published:** 2025-09-14

**Authors:** Sreevatsan Raghavan, Tanya Sarah Isaac, Divya Arya, Gabriela Cipriano Flores, Rekha Shanmugam, Ayushi, Bapu Koundinya Desiraju, Vidhya Venugopal, Ramachandran Thiruvengadam, Pallavi Kshetrapal, Nitya Wadhwa, Laura Downey, Jane E Hirst, Shinjini Bhatnagar, Heat in Pregnancy India (HiP- India) Group, TS Arya

**Affiliations:** 1 Translational Health Science and Technology Institute, Faridabad, Haryana, India; 2 Department of Environmental Health Engineering, Sri Ramachandra Institute of Higher Education and Research, Chennai, Tamil Nadu, India; 3 School of Public Health, Imperial College London, London, UK; 4 Biochemistry Department, Pondicherry Institute of Medical Sciences, Puducherry, Puducherry, India; 5 The George Institute for Global Health, Sydney, New South Wales, Australia; 6 The George Institute for Global Health UK, London, UK

**Keywords:** Epidemiology, Low and Middle Income Countries, Noncommunicable Diseases, Developing Countries

## Abstract

**Background:**

Preterm birth (PTB) is a major global health issue, causing substantial newborn morbidity and mortality. Limited literature is available on the association between prenatal exposure to heat and air pollution (particularly, particulate matter 2.5 (PM_2.5_) and black carbon) and the risk of PTB in low- and middle-income countries (LMICs). This review attempts to integrate existing information.

**Methods:**

A systematic search of numerous databases (Pubmed, Embase, Scopus and grey literature) for peer-reviewed articles published between 2010 and 2024 in LMIC was done using PM_2.5_, black carbon and ambient heat as exposures with PTB as the outcome. After screening 4524 studies, 32 were included, focusing on various exposure windows throughout pregnancy. The risk of bias assessment was done using the Non-randomised Studies of Exposures tool. Random-effects meta-analyses using the DerSimonian and Laird method were done when three or more studies were available; otherwise, a fixed-effects model was used to estimate the pooled effect sizes.

**Results:**

Heat exposures were associated with elevated incidences of PTB, especially during the first and third trimesters (OR=1.29 and 1.39, respectively). PM_2.5_ exposure was also similarly associated, but to a lower extent across all trimesters (OR=1.09). Black carbon exposure also depicted a similar trend, which was during the third trimester (OR=2.74).

**Conclusion:**

The results point towards a consistent adverse effect on the exposures studied (PM_2.5_, black carbon, and heat). There is also a dearth of representative data from LMICs where vulnerabilities to climate change, specifically for maternal and child health, are more pronounced. Furthermore, few studies have investigated the impact of combined exposures, highlighting a critical gap in understanding the synergistic effects of these environmental factors. This emphasises the need for more geographically diverse and representative studies to permit policy framing aiming to reduce PTB incidence mediated by environmental factors.

**PROSPERO registration number:**

The study protocol for this review was registered with PROSPERO-CRD42024563329.

**WHAT IS ALREADY KNOWN ON THIS TOPIC**

- Preterm birth (PTB) is a critical global health issue, particularly in low- and middle-income countries (LMICs), where environmental factors like heat and air pollution (particulate matter 2.5 (PM_2.5_) and black carbon) are known to contribute to adverse pregnancy outcomes. Previous research has often focused on high-income countries, resulting in inconsistent findings regarding the effects of these exposures on PTB, especially in LMICs where healthcare resources are limited and exposure levels may be higher.

**WHAT THIS STUDY ADDS**

- This is the first systematic review and meta-analysis to examine the combined impact of heat, black carbon and PM_2.5_ on pregnancy outcomes in LMICs.
- This study finds that heat exposure during the first and third trimesters is associated with a notable rise in PTB risk, while PM_2.5_ exposure shows a modest but significant effect across all trimesters. Notably, black carbon contributes to PTB through its dual role: direct health impacts and high radiative forcing, which exacerbates ambient temperatures and creates synergistic effects with heat exposure. The findings emphasise the compounded risks posed by simultaneous exposures to heat and air pollutants in hot and resource-limited settings.

**HOW THIS STUDY MIGHT AFFECT RESEARCH, PRACTICE OR POLICY**

- The findings underscore the urgent need for targeted public health interventions aimed at reducing environmental exposures during pregnancy in LMICs. Additionally, further research is essential to explore the mechanisms underlying these associations, their synergistic effects and the interaction between short-lived climate pollutants, such as black carbon and heat.

## Introduction

 Preterm birth (PTB), characterised as delivery prior to 37 completed weeks of gestation, continues to be a significant global health challenge, with 13.4 million estimated preterm live births each year.^1^ It is the leading cause of neonatal morbidity (which includes long-term physical complications and neurodevelopmental delays) and mortality, accounting for an estimated one million deaths annually.^2^ The incidence of PTB and the burden of PTB-associated neonatal mortality are more pronounced in low- and middle-income countries (LMICs), due to limited healthcare resources, inadequate medical infrastructure and heightened exposure to environmental stressors.^3^ The United Nations’ Sustainable Development Goal 3.2 seeks to significantly reduce neonatal and infant mortality by 2030 by lowering the neonatal mortality rate to 12 or fewer deaths per 1000 live births.

Environmental factors, particularly exposure to extreme heat and air pollution, contribute to adverse pregnancy outcomes, including PTB.^4^ The compounding risks from a rise in global temperatures due to climate change and elevated concentrations of air pollutants, particularly particulate matter 2.5 (PM_2.5_), are concerning for human health.^5^ Among these—black carbon (BC)—a component of PM_2.5_ resulting from incomplete combustion of fossil fuels and biomass poses a dual threat. First, its ability to penetrate deeply into the respiratory system and induce systemic effects, including oxidative stress and inflammation, makes it particularly harmful.^6^ Second, BC’s high radiative forcing contributes to increased ambient temperatures in its surrounding areas, exacerbating heat exposure.^7^ Exposure to extreme heat is hypothesised to induce maternal haemodynamic changes and disrupt placental function.^3^ This creates a synergistic effect in regions that are already experiencing persistently high temperatures, amplifying the risks to maternal and fetal health. These changes are thought to increase the risk of placentally mediated complications, such as PTB, intrauterine growth restriction, pre-eclampsia and stillbirth.

Although several reviews have examined the relationship between prenatal environmental exposures and pregnancy outcomes, findings have been inconsistent with varying effect sizes and directions, and there is often limited or no representative data from LMICs.^8^ These regions are often where the combined health impact of climate change is more severe. Most studies have considered heat and air pollution as independent factors, even though they frequently co-occur and may have synergistic effects on pregnancy outcome.^9^ With the ongoing challenges posed by climate change and air pollution, identifying and understanding these environmental determinants is essential towards developing effective strategies to reduce the global burden of PTB.

We aim to bridge these critical gaps by systematically reviewing and synthesising the available evidence on the association between prenatal exposure to heat and air pollution (PM_2.5_ and BC) and PTB in LMICs. The review attempts to answer: how do exposure to heat (ambient) and air pollution (PM_2.5_ and BC) in pregnancy impact the risk of PTB in LMICs? Additionally, it also aims to understand whether prenatal exposure to heat, PM_2.5_ and BC is associated with an increased risk of PTB in LMICs as separate and combined exposure entities.

## Materials and methods

Registration: this systematic review and meta-analysis has been registered in PROSPERO under the registration number CRD42024563329. Given the nature of this study, direct involvement of patients or the public in its design, execution, reporting or dissemination was neither possible nor appropriate.

### Search strategy

This systematic review and meta-analysis was conducted in accordance with the Preferred Reporting Items for Systematic Reviews and Meta-Analysis Protocols guidelines.^10^ The following databases were searched: EMBASE, PubMed, Scopus, Web of Science and Google Scholar. Only peer-reviewed original articles published in English were considered. The search was limited to articles published between 1 January 2010 and 20 June 2024, that is, the date the last search was performed and studies on humans.

We searched medical subject headings and Emtrees with keywords related to the exposures (heat, PM_2.5_ and BC) and PTB based on terminologies used in recent reviews on the topic.^4 9^ A comprehensive search strategy tailored to each database was consequently prepared, which can be found in online supplemental table 1. Selected key search terms for the heat, PM_2.5_ and BC exposures included (“ambient temperature” OR “heat strain” OR “heat exposure” OR “heat stress” OR “extreme heat “) and (“PM”, “PM_2.5_”, “particular matter”, “fine particular matter”, “black carbon”, “elemental carbon”, “elemental carbon particles”, “elemental carbon emissions”, “BC aerosols”), respectively. Select key search terms for the outcome included (“preterm birth”, “PTB”, “preterm delivery”, “premature labour” and “premature birth”). The search strategy was implemented with boolean combinations and modified according to each database where necessary. All search terms per exposure and outcome were connected using “OR” and combined the results with “AND”. After preliminary testing on the search terms, we modified and confirmed the retrieval strategies to suit the specific databases. Additionally, we conducted a manual search of reference lists of all included primary studies to identify any relevant publications.

### Patient and public involvement

No patients were involved.

### Study screening and selection

**Figure 1:**
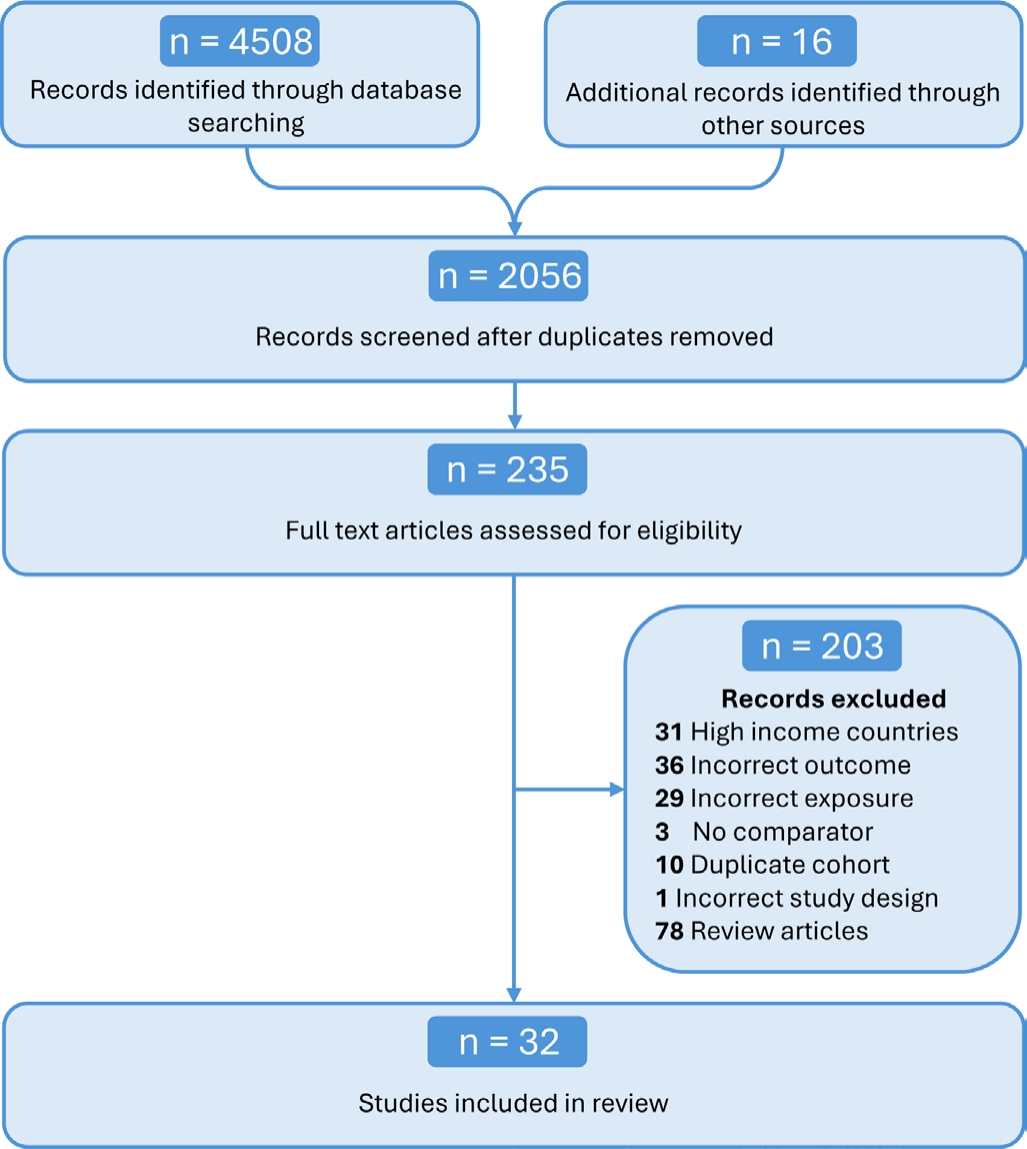
Preferred Reporting Items for Systematic Reviews and Meta-Analysis flow diagram of selection of studies in the meta-analysis.

The references of all the searches were exported to Covidence, a web-based platform for systematically conducting reviews, where all screening and data extraction took place. Four researchers independently screened the titles and abstracts of all articles found. Studies that did not examine the relationship between the specified exposures (heat, PM_2.5_, BC) and PTB were excluded. The remaining studies were deemed potentially relevant and underwent further review by four independent researchers. Studies were ultimately included in the meta-analysis if they fulfilled the specified eligibility criteria: (a) studies included any mention of heat, PM_2.5_ and/or BC exposures during pregnancy; (b) the outcome included PTB, defined as birth before the 37th week of gestation; (c) the study setting was a LMIC as defined by the World Bank at the time of the search^11^; (d) the study design had to be a quantitative observational study, encompassing cohort, case-control, cross-sectional, longitudinal or ecological studies; (e) participants were all above 18 years of age and (f) if there were multiple studies originating from overlapping samples, studies with larger sample sizes or longer durations were considered.^12^ Studies that did not qualify for the above criteria were excluded. A detailed overview of the study selection process is presented in figure 1.

### Quality assessment

The study design employed in this review encompassed cohort, time series and ecological methodologies. Four independent authors carried out the quality assessment following the Risk of Bias in Non-randomised Studies of Exposures tool. This is specifically designed to evaluate the risk of bias in observational studies that investigate the effects of exposures.^13^ Each study was evaluated across seven domains inclusive of confounding bias, participant selection, exposure assessment, deviations from intended exposures, missing data, outcome measurement and selection of reported results. Employing these evaluations, the studies were further classified as low, moderate, serious or critical risk of bias.^14 15^ All discrepancies were discussed and resolved by consensus to ensure the reliability and accuracy of the quality evaluation.

### Data extraction

Four researchers independently extracted data, and any discrepancies were discussed and resolved by consensus. Each researcher used a standardised extraction table to extract all pertinent information from the selected studies. The following data was extracted: authors, title, publication year, country, site type, sample size, percentage of PTB cases, duration of data collection, study design, sample type, source of outcome data, outcome definition, outcome types, PTB subtypes, exposure data source, exposure windows, exposure category, exposure definition, statistical method used and effect estimate: OR (odds ratio), RR (relative risk), or HR (hazard ratio), and their 95% CIs) reported. Two independent reviewers resolved discrepancies by individually voting in favour or against the article in dispute. The article was excluded or included on the basis of this voting.

### Statistical analysis

Random effects meta-analyses (DerSimonian and Laird) were only performed if at least three studies reported the association between the exposure and outcome within the same exposure window; otherwise, a fixed effects model was used. Two sets of random effects meta-analyses were conducted: for heat (barring the entire pregnancy exposure window) and PM_2.5_ exposures in various exposure window groups. OR was used as the primary indicator of the overall effect. We assumed that the HR approximates the risk ratio since the prevalence of PTB in most of our studies was below 10%. Therefore, the same formula was used to convert both RRs and HRs to ORs.^15^

In order to facilitate the comparison of effect sizes from various studies, units across studies were aggregated based on a standardised increase in pollutant concentration of 10 µg/m^3^ for PM_2.5_. Standardised risk estimates were computed for each study with the subsequent formula:

EE₁₀ = EE^((10/Δx))^,

where:

- EE₁₀ is the standardised effect estimate for a 10 µg/m³ increase in PM_2.5_.
- EE is the reported estimate (or OR, HR, etc) from the study.
- Δx is the unit of concentration increase (in μg/m³) used in the original study.

This method adjusts each study’s effect size to reflect the relative risk associated with a 10 µg/m³ increment in PM_2.5_.

The DerSimonian and Laird method accounts for both within-study and between-study variability, which is critical when studies exhibit heterogeneity. The formula for pooling the ORs is based on the inverse variance method, where the weight assigned to each study is the inverse of the variance of its estimated effect size. I^2^ statistics were employed to assess the studies’ heterogeneity. An I^2^ value below 50% suggested low or moderate heterogeneity between studies, for which a fixed effects model was applied. When I^2^ ≥50%, indicating substantial heterogeneity, a random effect model was implemented.^16^ Publication bias was assessed using funnel plots and Egger tests.^14^ Egger’s test, with a p value<0.05 indicating the presence of publication bias. Sensitivity analyses were performed to explore the impact of study quality and sample size on the pooled estimates via ‘trim and fill’ and ‘leave-one-out’ methods to assess publication bias and the impact of individual studies on the overall effect estimates. All analyses were conducted in R (V.4.3.1) using the ‘meta’ (V.8.0–0) and ‘metafor’ (V.4.7–26) packages.

## Results

### Study characteristics

The literature search across five databases yielded 4508 articles, with an additional 16 identified from reference lists. After removing 2468 duplicates, 2056 titles and abstracts were screened, leaving 235 full texts for review, with a final total of 32 articles being included for analysis. Nine articles studied the association between maternal heat exposure and PTB.^17^,^25^ 20 articles studied the impact of PM_2.5_ exposure on PTB.^26^,^3232^ Two articles explored the effects of maternal BC exposure on PTB.^45 46^ One explored the combined effect of heat and PM_2.5_ exposure on PTB.^47^

Notably, 28 of the studies were conducted in China, with a select few from South Asia and Africa, which limits the generalisability of findings to other LMICs. Sample sizes ranged from 656 to over 2 million participants, with cohort studies being the predominant study design. Exposure timing varied significantly, from trimester-specific periods to the entire pregnancy duration. PTB was consistently defined as delivery before 37 weeks of gestation and also distinguished between spontaneous and medically induced PTB. Adjustments for confounding factors varied, with some studies accounting for socioeconomic status, maternal health and environmental factors, while others used more limited adjustments. This heterogeneity in study designs, exposure timing and confounding adjustments must be considered when interpreting the pooled results. The contextual summary for the same has been presented in online supplemental table 2 and the risk of bias assessment summary has been illustrated in table 1.

**Table 1:** Risk of bias assessment summary for the studies included in the review using the ROBINS-E tool

First author	Country	Risk of bias - confounding	Risk of bias - measurement of exposure	Risk of bias - selection of participants	Risk of bias - measurement of outcomes	Risk of bias - reported result
Wang *et al*^21^	China	Low risk	Some concerns	Low risk	Low risk	Low risk
Zheng *et al*^25^	China	Low risk	Low risk	Low risk	Low risk	Low risk
Weng *et al*^23^	China	Low risk	Some concerns	Low risk	Low risk	Low risk
Wu *et al*^24^	China	Low risk	Low risk	Some concerns	Low risk	Low risk
Wang *et al*^22^	China	Low risk	Some concerns	Low risk	Low risk	Low risk
Shankar *et al*^20^	India and Pakistan	Some concerns	Low risk	Low risk	Low risk	Low risk
Qian *et al*^35^	China	Some concerns	Low risk	Low risk	Low risk	Low risk
Li *et al*^30^	China	Some concerns	Low risk	Low risk	Low risk	Low risk
*Liu et al* ^31^	China	Low risk	Low risk	Some concerns	Low risk	Low risk
Li *et al*^29^	China	Some concerns	High risk	Low risk	Low risk	Low risk
Liu *et al*^32^	China	Low risk	High risk	Some concerns	Low risk	Low risk
Sun *et al*^37^	China	Some concerns	Low risk	Low risk	Low risk	Low risk
Wang et al^17^	China	Low risk	Low risk	Low risk	Low risk	Low risk
Yuan *et al*^41^	China	Low risk	Low risk	Low risk	Low risk	Low risk
Chen *et al*^27^	China	Low risk	Low risk	Low risk	Low risk	Low risk
Wang *et al*^47^	China	Some concerns	Some concerns	Low risk	Low risk	Low risk
Tapia *et al*^38^	Peru	Low risk	Some concerns	Low risk	Low risk	Low risk
Li *et al*^44^	China	Some concerns	Low risk	Low risk	Low risk	Low risk
Zhou *et al*^43^	China	Low risk	Low risk	Low risk	Low risk	Low risk
Zhou *et al* ^48^	China	Low risk	Low risk	Low risk	Low risk	Low risk
Fang *et al*^45^	China	Low risk	Low risk	Low risk	Low risk	Low risk
He *et al*^28^	China	Low risk	Low risk	Low risk	Low risk	Low risk
Wang *et al*^39^	China	Low risk	Some concerns	Low risk	Low risk	Low risk
He *et al*^46^	China	Low risk	Low risk	Low risk	Low risk	Low risk
Li *et al*^40^	China	Low risk	Some concerns	Low risk	Low risk	Low risk
Ming *et al*^33^ 2023	China	Some concerns	Low risk	Low risk	Low risk	Low risk
Qiu *et al*^36^ 2023	China	Low risk	Low risk	Low risk	Low risk	Low risk
Mitku *et al*^34^ 2023	South Africa	Some concerns	Low risk	Low risk	Low risk	Low risk
Yu *et al*^42^ 2024	China	Low risk	Low risk	Low risk	Low risk	Low risk
Zhang *et al*^19^ 2024	China	Low risk	Low risk	Low risk	Low risk	Low risk
Bachwenkizi *et al*^26^ 2022	15 African countries (Tanzania, Cameroon, Zimbabwe, Mali, Nigeria, Chad, Benin, South Africa, Burundi, Uganda, Ethiopia, Guinea, Zambia, Angola and Malawi)	Low risk	Some concerns	Low risk	Low risk	Low risk
He *et al*^18^ 2016a	China	Low risk	Low risk	Some concerns	Low risk	Low risk

ROBINS-E, Risk of Bias in Non-randomised Studies of Exposures.

### Heat exposure

**Figure 2:**
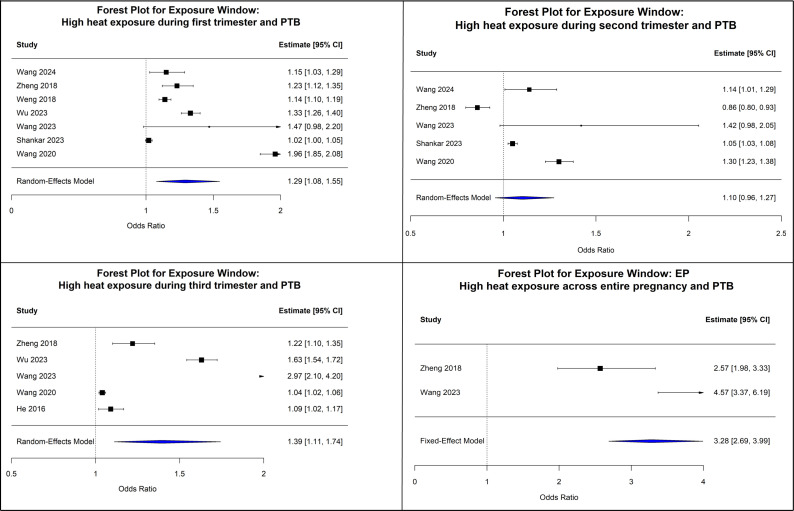
Forest plots of meta-analysis in the associations between heat-exposed trimesters and preterm birth (PTB) (each trimester: T1, T2, T3, respectively, and the entire pregnancy (EP)).

Of the studies exploring the association between heat exposure and PTB, seven reported exposures in the first trimester, five in the second trimester and five in the third trimester, with two studies reporting the effect of average exposure across the entire pregnancy. Across all analyses, heterogeneity was very high (I² >95% for all three trimesters). Due to the high heterogeneity present between the studies, the Der Simonian and Laird random effects model was applied for the three individual trimesters, whereas a fixed effects model was used for studies covering the entire pregnancy period, as only two studies were available. There was a significant increase in the risk of PTB with heat exposure in the first trimester (OR=1.29; 95% CI: 1.08 to 1.55) and third trimester (OR=1.39; 95% CI: 1.11 to 1.74); however, there was a non-significant association for exposure in the second trimester (OR=1.1; 95% CI: 0.96 to 1.27). There was a three-fold increase in the risk of PTB with high heat exposure across the entire pregnancy (OR=3.28; 95% CI: 2.99 to 3.99), suggesting that heat exposure across the entire pregnancy was associated with a markedly elevated risk of PTB. Egger’s test indicated no publication bias for first-trimester, second-trimester and third-trimester heat exposure (p>0.05). The Tau^2^ test could not be conducted for the entire pregnancy since only two articles provided point estimates for this exposure window. The forest plots for the same are depicted in figure 2. A summary of the review results, including the association estimates, heterogeneity tests and Egger’s test for heat, PM_2.5_ and BC exposure in relation to PTB, is presented in table 2. Additionally, more detailed descriptions of the studies included in the review with the outcome of interest are available in online supplemental table 2.

**Table 2:** Summary of review results of association, heterogeneity test and Egger’s test between heat and air pollution (PM_2.5_ and black carbon) and preterm birth

Exposure	Trimester	No. of studies	Test of association		I^2^ (%)	Test of heterogeneity		Tau^2^	P value_Egger’s_
			OR	95% CI		Q	P value		
Heat	First trimester	7	1.29	1.08 to 1.55	98.6	441.35	<0.0001	0.055	0.628
	Second trimester	5	1.10	0.96 to 1.27	95.1	81.96	<0.0001	0.022	0.336
	Third trimester	5	1.39	1.11 to 1.74	98.4	250.23	<0.0001	0.06	0.004
	Entire pregnancy	2* (fixed effects model used)	3.28	2.69 to 3.99	N/A	7.96	0.005	–	0.006
PM_2.5_	First trimester	9	1.02	0.99 to 1.06	96.7	243.47	<0.0001	0.002	0.117
	Second trimester	12	1.04	1.00 to 1.07	97.8	506.66	<0.0001	0.002	0.875
	Third trimester	11	1.06	1.03 to 1.09	98	511.54	<0.0001	0.002	0
	Entire pregnancy	19	1.09	1.06 to 1.13	99.2	2338.01	<0.0001	0.004	0.177
BC	First trimester	2	1.11	1.07 to 1.14	2.07	1.021	0.312	0.0003	–
	Second trimester	1	1.11	0.83 to 1.47	–	–	–	–	–
	Third trimester	1	2.74	2.86 to 4.05	–	–	–	–	–
Heat and PM_2.5_	First trimester	1	1.23 1.73	1.09 to 1.37 1.57 to 1.91	–	–	–	–	–
	Second trimester	1	0.97 1.17	0.86 to 1.08 1.06 to 1.29	–	–	–	–	–
	Third trimester	1	1.44 0.94	1.29 to 1.61 0.84 to 1.04	–	–	–	–	–
	Entire pregnancy*	2	Heat: 1.48 PM_2.5_: 1.70 Heat and PM_2.5_: 1.92	1.31 to 1.67 1.59 to 1.81 1.39 to 3.64	–	–	–	–	–

Test of heterogeneity was not conducted for three studies. Egger’s test was not conducted for five studies.

* Although there were two studies for this exposure window, heterogeneity tests could not be conducted as one study reported the OR of combined exposures and the second reported individual ORs for each exposure.

BC, black carbon; PM, particulate matter.

### PM_2.5_ exposure

**Figure 3:**
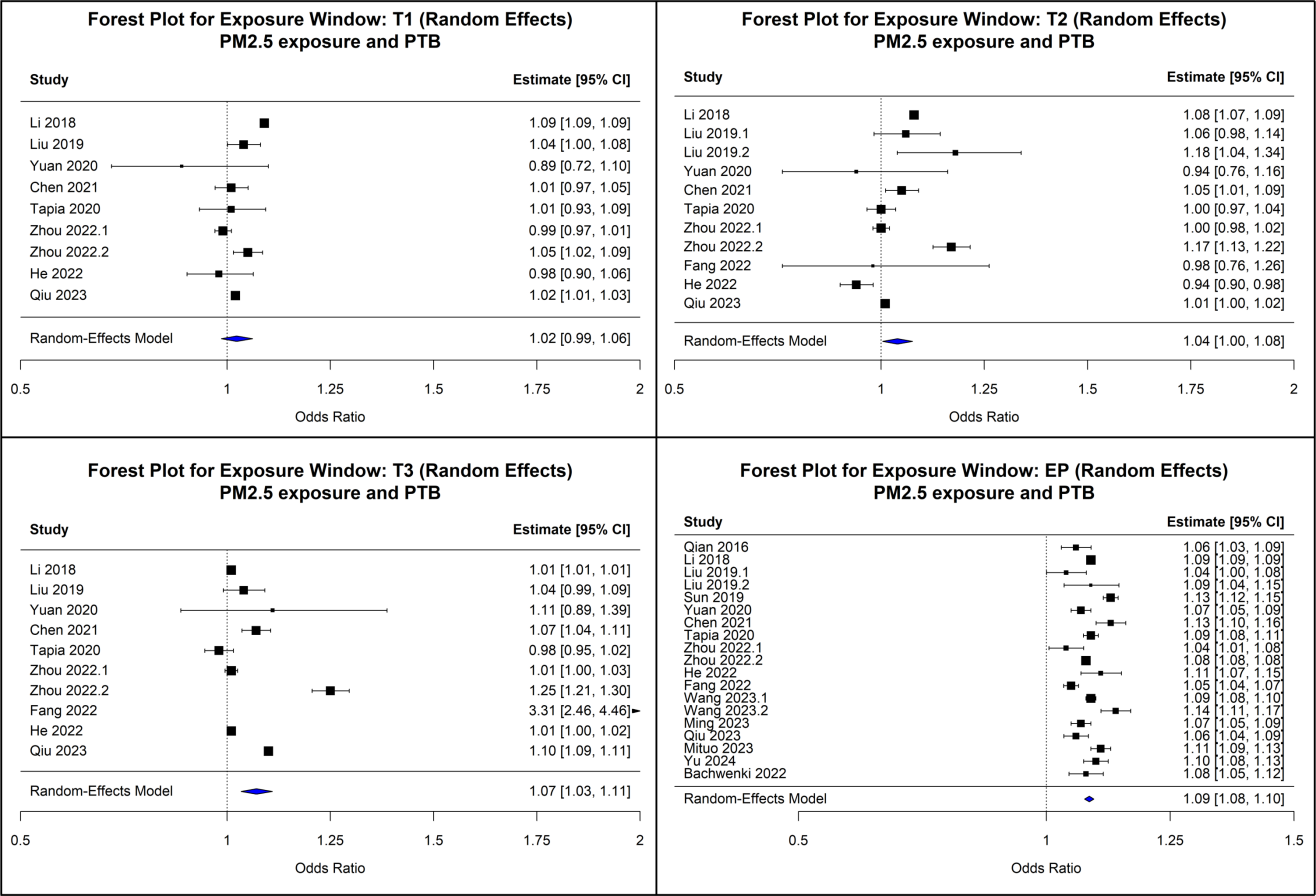
Forest plots of meta-analysis in the associations between particulate matter 2.5 (PM_2.5_) exposed trimesters and preterm birth (PTB) (each trimester- T1, T2, T3, respectively, and the entire pregnancy (EP)).

We observed an increase in risk of PTB with PM_2.5_ exposure; however, the effect size was smaller than for heat. In the first trimester, nine studies showed a weak association (OR=1.02; 95% CI: 0.99 to 1.06; I² = 96.7%). In the second trimester, 12 studies revealed a marginally significant increase in risk (OR=1.04; 95% CI: 1.00 to 1.07, I² = 97.8%). The third trimester presented a similarly modest association, with an OR of 1.06 (95% CI: 1.03 to 1.09, I² = 98%). Across the entire pregnancy, 19 studies reported a modest but statistically significant effect (OR=1.09; 95% CI: 1.06 to 1.13, I² = 99.2%). Egger’s test indicated no publication bias across all exposure windows (p>0.05). Figure 3 presents the forest plots illustrating these associations.

### BC exposure

We found a significant association between BC exposure and an elevated risk of PTB in the third trimester; however, the results were mixed across trimesters one and two. Studies demonstrated a significant association for first trimester BC exposure (OR=1.11; 95% CI: 1.07 to 1.14; I² = 2.07%). However, one study on second-trimester BC exposure reported a non-significant association (OR=1.11, 95% CI: 0.83 to 1.47). Notably, the strongest effect was observed in the third trimester, with one study reporting a substantial increase in PTB risk (OR=2.74, 95% CI: 2.86 to 4.05). Due to the limited number of studies, heterogeneity was low to moderate, and Egger’s test could not be performed. Sensitivity analyses were not applicable.

### Heat and PM_2.5_ exposure

Exposure to both PM_2.5_ and heat increased the risk of PTB, while only based on two studies, the effect direction was consistent. We observed a significant combined effect of heat and PM_2.5_ exposure on PTB across the entire pregnancy, with one study reporting an OR of 1.92 (95% CI: 1.39 to 3.64). Another study examined the individual effects of both exposures, showing that heat exposure throughout the entire pregnancy had an OR of 1.48 (95% CI: 1.13 to 1.67), while PM_2.5_ exposure resulted in a higher OR of 1.70 (95% CI: 1.59 to 1.81). Due to the limited number of studies, publication bias tests could not be performed, and sensitivity analyses were not applicable.

### Sensitivity analysis

Both sensitivity analyses for heat and PM_2.5_ revealed that while the heterogeneity was high, there was minimal publication bias for the three-trimester exposure windows. The effect estimates assessing the entire pregnancy period as an exposure window were deemed to be underestimated, as there could be some missing studies in that particular window for both heat and PM_2.5_. The ‘leave-one-out’ sensitivity analysis also highlighted that the associations observed across all exposure windows were relatively robust, with no single study biasing the same in either direction, despite the high level of heterogeneity. The results of which are presented in online supplemental tables 3 and 4. Results of the ‘trim-and-fill’ sensitivity analysis are presented in online supplemental figures 1–4.

## Discussion

### Key findings

This systematic review and meta-analysis examined 32 studies to investigate the associations between maternal exposure to ambient temperature (heat) and air pollution (PM_2.5_ and BC) at different gestational periods and the risk of PTB, focusing solely on LMICs. The strongest association was observed between exposure to heat across the entire pregnancy duration and PTB; however, this effect estimate was based on two studies from China. These findings align with the growing body of evidence regarding the risks of environmental exposures on birth outcomes.

This review’s pooled effect sizes are higher than those reported in similar studies from high-income countries (HICs), likely reflecting the intersection of multiple stressors in LMICs such as extreme heat, poor air quality and limited resources for mitigating these exposures.^4^ Pregnant women in these regions are often exposed to both higher levels of air pollution and extended periods of extreme heat, likely contributing to the increased risk of PTB.^47^

The high level of heterogeneity observed across studies in this review may be attributed to several factors, including differences in geographic regions, study designs and exposure measurement methods, thus highlighting the variability in environmental conditions and maternal exposure patterns in LMICs. Two contributing factors pertinent to LMICs include urban-rural disparities in exposure levels^29^ and PM_2.5_ composition regional variability. Urban populations are at risk of significantly higher PM_2.5_ and heat exposure compared with rural populations, which is exacerbated due to the urban heat island effect.^1^ PM_2.5_ composition tends to vary regionally due to local factors, such as industrial activities, sources of emissions and climatic conditions. Therefore, some reasons might have high concentrations of BC and heavy metals, while others might primarily derive PM_2.5_ from biomass burning. Both urban-rural disparities and regional variability of PM_2.5_ composition and their associated effects on PTB remain relatively unexplored, highlighting the need for further investigation.^20 26^

### Comparison with previous meta-analyses on heat and PM_2.5_ exposure

Our findings align with previous meta-analyses analysing the relationship between heat and PM_2.5_ exposure and PTB; however, several differences set this study apart. Notably, previous meta-analyses, such as those by Bekkar *et al* and Chersich *et al*, primarily focused on HICs, particularly in North America and Europe, where air quality monitoring systems for environmental pollutants are robust and healthcare infrastructure is stronger.^4^ These studies generally reported smaller effect sizes, likely because HICs have more effective mechanisms in place to mitigate the impact of heat and air pollution.

Moreover, our meta-analysis incorporated a broader range of exposure assessments, including central site monitoring, personal monitoring and model-based approaches (such as pollutant dispersion modelling and land-use regression), providing a broader perspective on how different approaches to exposure measurement influence outcomes and reduce bias.

### Strengths and limitations

A major strength of this study is its status as the most up-to-date systematic review and meta-analysis on the topic of combined ambient heat, BC and PM_2.5_ exposure during pregnancy and their associations with PTB, with data solely from LMICs. The meta-analysis approach used in this study not only allows for a more robust analysis (despite the high heterogeneity) by pooling results from diverse settings. By including trimester-specific and cumulative exposures, the study provides a detailed understanding of how these environmental factors can affect different stages of pregnancy. The rigorous inclusion criteria and comprehensive statistical analysis also enhance the credibility of the findings.

In the same stance, the authors also acknowledge certain limitations. First, a meta-regression for BC and cumulative exposure to heat and PM_2.5_ was not done due to insufficient studies. Second, the majority of the studies were geographically centred in China, which may affect the generalisability of the results. However, the authors opine that this also underscores the need for more representative studies from the LMIC region. Third, high heterogeneity was observed, which may be due to several reasons, such as unmeasured variables, such as the specific components of PM_2.5_ and interactions with other pollutants and urban-rural disparities etc. Another reason for the high heterogeneity may be due to the varying data sources from which ambient temperature data were derived, which ranged from satellite-based measurements, meteorological data websites, regional meteorological data centres or local weather stations. Fourth, most studies focused on either heat or PM_2.5_ in isolation, making it challenging to disentangle their combined effects and control for confounding. The effect of heat on PTB might be partially due to the presence of PM_2.5_, particularly BC, a short-lived climate pollutant known to increase ambient temperature, as both often occur together and interact in complex ways.

### Implications for clinicians and policymakers

In addition to quantifying the risk, this review also attempts to explore the potential of the compounded effects of simultaneous exposure to heat and air pollution. Given that these environmental stressors frequently interact in real-world circumstances, it is critical to understand their combined impact on PTB risk. Heat exposure can increase the consequences of air pollution by raising metabolic demand and the vulnerability of the cardiovascular and respiratory systems.^29^ This study seeks to give insights into the interaction of these exposures, which could guide public health interventions and policy initiatives geared to lessen the impact of environmental stressors on pregnancy outcomes in LMICs.

The authors highlight that although the studies are limited, the findings have the potential to inform public health policy and interventions in LMICs. The review not only underscores the need for more diverse and representative research but also aims to highlight the need for targeted policies to reduce exposure to extreme heat and air pollution. Such initiatives, which may include those mentioned in Heat Action Plans, must address the impact of both heat and air pollution via urban infrastructure and design modifications and the establishment of heat warning systems.^5^ Expansion of greenspace in urban areas will mitigate the effects of urban heat islands and has also been found to reduce PM_2.5_ exposure in pregnant women, potentially lowering the risk of not only PTB but other adverse pregnancy outcomes as well.^29^

### Future perspectives

Many important research questions remain unanswered. For instance, future research priorities could include exploring alternative exposure metrics and the identification of critical windows of exposure and examining underlying pathophysiological mechanisms. Additionally, studies should aim to clarify the combined effects of air pollution and heat, particularly in regions outside of China where environmental conditions and healthcare systems differ, elaborating on the pathophysiological mechanisms behind the same as well. More research is required to investigate how PM_2.5_ components, such as BC, contribute to PTB risk, especially in relation to different pollutant compositions across regions.

There also needs to be more geographical diversity and inclusivity in this realm of research, where varying environmental and social determinants contribute to the observed risks. This will help us better understand how the compounded risk of heat and air pollution varies regionally and develop more tailored interventions.

## Conclusion

In this systematic review and meta-analysis of 32 studies, we explored the associations between maternal exposure to ambient heat and air pollution during different gestational periods and the risk of PTB, focusing on LMICs. Our findings indicate that heat exposure, particularly across the entire pregnancy, is linked to an increased risk of PTB, with a pooled effect size greater than those reported in HICs, underscoring the compounded challenges faced by pregnant women in LMICs. Further inclusive quality research will help in shaping policy decisions and public health interventions.

## Supplementary material

10.1136/bmjpo-2025-003428 online supplemental file 1

## Data Availability

Data are available upon reasonable request.
