# Supplementary material for: Association between heat and air pollution (PM2.5 and black carbon) exposure in pregnancy and preterm birth in low- and middle-income countries: a systematic review and meta-analysis
Source: BMJ Paediatr Open. 2025 Sep 14;9(1):e003428. doi: 10.1136/bmjpo-2025-003428 (PMC12434743; doi:10.1136/bmjpo-2025-003428)

**Supplementary Tables and Figures**

**Supplementary Table 1:** Search strategy

| Sl No. | Database | Search String |
| --- | --- | --- |
| 1 | PubMed | ("premature labour" OR "premature birth" OR "pregnancy complications" OR "pregnancy outcomes" OR "adverse pregnancy outcomes" OR "pre-term birth") AND ("air pollutants" OR "air pollution" OR "coal power" OR "fine particulate" OR "pm 2.5" OR "fine particle" OR "black carbon" OR "elemental carbon" OR "vehicular exhaust" OR "vehicle emissions" OR "diesel emissions" OR "biomass burning" OR "indoor air pollution" OR "household air pollution") AND ("ambient temperature" OR "heat exposure" OR "heat stress" OR "heat wave" OR "climate change" OR "global warming") AND ("socio-economic status" OR "socio-economic factors" OR "low- and middle-income countries" OR "LMICs") |
| 2 | Scopus | TITLE-ABS-KEY(("premature labour" OR "premature birth" OR "pregnancy complications" OR "pregnancy outcomes" OR "adverse pregnancy outcomes" OR "pre-term birth") AND ("air pollutants" OR "air pollution" OR "coal power" OR "fine particulate" OR "pm 2.5" OR "fine particle" OR "black carbon" OR "elemental carbon" OR "vehicular exhaust" OR "vehicle emissions" OR "diesel emissions" OR "biomass burning" OR "indoor air pollution" OR "household air pollution") AND ("ambient temperature" OR "heat exposure" OR "heat stress" OR "heat wave" OR "climate change" OR "global warming") AND ("socio-economic status" OR "socio-economic factors" OR "low- and middle-income countries" OR "LMICs")) |
| 3 | Web of Science | TOPIC: ("premature labour" OR "premature birth" OR "pregnancy complications" OR "pregnancy outcomes" OR "adverse pregnancy outcomes" OR "pre-term birth") AND ("air pollutants" OR "air pollution" OR "coal power" OR "fine particulate" OR "pm 2.5" OR "fine particle" OR "black carbon" OR "elemental carbon" OR "vehicular exhaust" OR "vehicle emissions" OR "diesel emissions" OR "biomass burning" OR "indoor air pollution" OR "household air pollution") AND ("ambient temperature" OR "heat exposure" OR "heat stress" OR "heat wave" OR "climate change" OR "global warming") AND ("socio-economic status" OR "socio-economic factors" OR "low- and middle-income countries" OR "LMICs") |
| 4 | EMBASE | ('premature labour'/exp OR 'premature birth'/exp OR 'pregnancy complication'/exp OR 'pregnancy outcome'/exp OR 'adverse pregnancy outcome'/exp OR 'preterm birth'/exp) AND ('air pollutant'/exp OR 'air pollution'/exp OR 'coal'/exp OR 'fine particulate matter'/exp OR 'pm 2.5'/exp OR 'fine particle'/exp OR 'black carbon'/exp OR 'elemental carbon'/exp OR 'vehicular exhaust'/exp OR 'vehicle emission'/exp OR 'diesel exhaust'/exp OR 'biomass burning'/exp OR 'indoor air pollution'/exp OR 'household air pollution'/exp) AND ('ambient temperature'/exp OR 'heat exposure'/exp OR 'heat stress'/exp OR 'heat wave'/exp OR 'climate change'/exp OR 'global warming'/exp) AND ('socioeconomic status'/exp OR 'socioeconomic factor'/exp OR 'low income country'/exp OR 'middle income country'/exp OR 'developing country'/exp) |
| 5 | Google Scholar | intitle:"premature labour" OR intitle:"premature birth" OR intitle:"pregnancy complications" OR intitle:"pregnancy outcomes" OR intitle:"adverse pregnancy outcomes" OR intitle:"pre-term birth" AND ("air pollutants" OR "air pollution" OR "coal power" OR "fine particulate" OR "pm 2.5" OR "fine particle" OR "black carbon" OR "elemental carbon" OR "vehicular exhaust" OR "vehicle emissions" OR "diesel emissions" OR "biomass burning" OR "indoor air pollution" OR "household air pollution") AND ("ambient temperature" OR "heat exposure" OR "heat stress" OR "heat wave" OR "climate change" OR "global warming") AND ("socio-economic status" OR "socio-economic factors" OR "low- and middle-income |

## Supplementary Table 2: Summary table for the studies included in the review with the outcome of interest as preterm birth

| **S.no** | First Author, Year of publication | Location | Study Period | Study Design | Sample Size | PTB rate (%) | Exposure assessed | Exposure Period |
| --- | --- | --- | --- | --- | --- | --- | --- | --- |
| 1. | Wang 2024 | China | 2015–2018 | Cohort study | 197080 | 3.8 | Heat (satellite-based quantification) | Entire Pregnancy |
| 2. | Zheng 2018 | China | September 2011–January 2012 | Cross sectional study | 3604 | 4.0 | Heat (meteorological website- wunderground.com) | Conception month, three trimesters, birth month and entire pregnancy |
| 3. | Weng 2018 | China | January 2001–December 2010 | Cross sectional study | 2123751 | 6.62-7.57 | Heat (Weather monitoring stations) | At delivery |
| 4. | Wu 2023 | China | January 2010–December 2018 | Cohort study | 205771 | 8.75 | Heat/Heat Stress (data sourced from China National Meteorological Information Center) | 1 week of pregnancy, 4 weeks of pregnancy, 4 weeks before delivery, and 1 week before delivery |
| 5. | Wang 2023 | China | March 2013– December 2018 | Cohort study | 10826 | 3.71 | Heat (data sourced from China National Meteorological Information Center, fine-scaled to 1X1 km resolution) | Three trimesters, the entire pregnancy, one week and four weeks before delivery |
| 6. | Shankar 2023 | India, Pakistan | 2014–2020 | Cohort study | 126273 | 16.4 | Heat (sourced from automated observation systems) | At enrolment during pregnancy, within 72 hours of delivery and at 42 days postpartum |
| 7. | Qian 2016 | China | June 10, 2011–June 9, 2013 | Cohort study | 95911 | 4.5 | PM_2.5_ | 7-14 days pre-conception, each pregnancy month, every trimester, entire pregnancy duration |
| 8. | Li 2018 | China | December 1st, 2013- November 30th, 2014 | Cohort study | 1240978 | 8.1 | PM_2.5_ | Every trimester of pregnancy |
| 9. | Liu 2019 | China | January 1, 2014–December 31, 2015 | Cohort study | 86139 | 0.79 | PM_2.5_ | Each trimester of pregnancy |
| 10. | Li 2020 | China | 2014 | Cross sectional | 429865 | 2.98 | PM_2.5_ | Entire pregnancy duration |
| 11. | Liu 2019 | China | 2016–2019 | Cohort study | 4101 | 5.7 | PM_2.5_ | First trimester onwards till delivery |
| 12. | Sun 2019 | China | 2013-2017 | Cross sectional study | 6275 | 5.9 | Heat (derived from ground monitoring stations), PM_2.5_ | Each pregnancy month, trimester, and entire pregnancy duration |
| 13. | Wang 2020 | China | January 2015 - July 2017 | Cross Sectional | 215059 | 5.17 | Heat (derived from local monitoring stations), PM_2.5_ | Final Gestational week |
| 14. | Yuan 2020 | China | 2013-2016 | Cohort study | 3692 | 4.6 | PM_2.5_ | Each trimester of pregnancy |
| 15. | Chen 2021 | China | 2015-2017 | Cohort study | 13111 | 4.68 | PM_2.5_ | Every trimester of pregnancy |
| 16. | Wang 2020 | China | 2013-2014 | Cohort study | 1281859 | 8.1 | Heat (sourced from China Meteorological Data Sharing Service System) | Every trimester of pregnancy |
| 17. | Tapia 2020 | Peru | 2012-2016 | Cross sectional study | 123034 | 6.5 | PM_2.5_ | Average exposure during each trimester and the entire pregnancy |
| 18. | Li 2021 | China | January 1, 2016 - June 30, 2019 | Cross sectional study | 120446 | 4.48 | PM_2.5_ | Average weekly exposure per trimester of pregnancy |
| 19. | Zhou 2022 | China | 2015-2020 | Cohort study | 572116 | 5.88 | PM_2.5_ | Mean concentrations during the entire pregnancy and every trimester |
| 20. | Zhou 2022 | China | 2014-2016 | Cross sectional study | 679316 | 2.2 | PM_2.5_ | Mean concentrations during the entire pregnancy and every trimester |
| 21. | Fang 2022 | China | 2014-2017 | Cross-sectional study | 24001 | 4.4 | PM_2.5_, Black Carbon | The entire duration of the pregnancy |
| 22. | He 2022 | China | January 2010 - December 2015 | Cohort study | 3723139 | 7.7 | PM_2.5_, Black Carbon | Each trimester, and entire pregnancy duration |
| 23. | Wang 2022 | China | June 2018 - November 2019 | Cohort study | 424 | 4.00 | PM_2.5_ | Entire duration of pregnancy |
| 24. | He 2022 | China | January 2018 -December 2019 | Cohort study | 10722 | 4.29 | PM_2.5_ | Each trimester, and entire pregnancy duration |
| 25. | Wang 2023 | China | January, 2018 – December 2019 | Cohort study | 6133 | 6.9 | PM_2.5_ | Each trimester, and entire pregnancy duration |
| 26. | Ming 2023 | China | 2015–2020 | Cross-Sectional study | 598018 | 5.86 | PM_2.5_ | Entire duration of pregnancy |
| 27. | Qiu 2023 | China | 2013-2019 | Cohort study | 2294188 | 3.61 | PM_2.5_ | Each trimester, and entire pregnancy duration |
| 28. | Mitku 2023 | South Africa | 2013–2017 | Cohort study | 656 | 17.22 | PM_2.5_ | Entire pregnancy duration. |
| 29. | Yu 2024 | China | 2011-2021 | Cohort study | 61762 | 3.19 | PM_2.5_ | Each trimester, and entire pregnancy duration, and 180 days prior to conception |
| 30. | Zhang 2024 | China | 2019-2020 | Cross sectional study | 34365 | 5.74 | Heat (data sourced from Shandong Environmental Information Monitoring Center and the China Meteorological Science Data Sharing Service), PM_2.5_ | Each trimester, and entire pregnancy duration |
| 31. | Bachwenkizi 2022 | 15 African countries (Tanzania, Cameroon, Zimbabwe, Mali, Nigeria, Chad, Benin, South Africa, Burundi, Uganda, Ethiopia, Guinea, Zambia, Angola, and Malawi) | 2005–2015 | Cohort study | 131594 | 3.3 | PM_2.5_ | Entire duration of pregnancy |
| 32. | He 2016 | China | 2001–2011 | Cross-sectional study | 838146 | 5.6 | Heat (sourced from the Guangzhou Meteorological Bureau) | The last week of pregnancy, the last 4 weeks of pregnancy, late pregnancy (gestational week 20 onward), and the entire pregnancy |

**Supplementary Table 3:** **In-depth analysis of** the studies included in the review with the outcome of interest as preterm birth

| S.no | First Author, Year of publication | Title | Location | Study Period | Study Design | Sample Size | PTB rate (%) | Exposure assessed | Exposure Period | Adjustments during analysis |
| --- | --- | --- | --- | --- | --- | --- | --- | --- | --- | --- |
| 1. | Wang 2024 | Heat exposure-induced risks of preterm birth mediated by maternal hypertension | China | 2015–2018 | Cohort study | 197080 | 3.8 | Heat (Daily meteorological data interpolated to 1 X 1 km using inverse distance weighting using satellite based imaging) | Entire Pregnancy | (1) maternal demographic characteristics: age (continuous), pre-pregnancy body mass index (BMI) (continuous), education residential area, season of conception behavioral risk factors and Adequacy of Prenatal Care Utilization (APNCU) index categories); (2) fetal variables: year of birth, parity and infant sex; and (3) environmental factors: cold exposure during 1–20 weeks, average temperature after 20 weeks of gestation, relative humidity and air pollution exposure (continuous) during the entire pregnancy |
| 2. | Zheng 2018 | An epidemiological assessment of the effect of ambient temperature on the incidence of preterm births: Identifying windows of susceptibility during pregnancy | China | September 2011–January 2012 | Cross sectional study | 3604 | 4.0 | Heat (Daily temperature data from meteorological website- wunderground.com) | Conception month, three trimesters, birth month and entire pregnancy | Fetal sex, birth season, birth year, maternal reproductive age, parental socioeconomic status (SES) indicated by house size), and indoor factors(environmental tobacco smoke (ETS), new furniture and redecoration, visible mold/damp stains, ventilation condition or window condensation in winter, and household pets during pregnancy |
| 3. | Weng 2018 | Adverse neonatal outcomes in relation to ambient temperatures at birth: A nationwide survey in Taiwan | China | January 2001–December 2010 | Cross sectional study | 2123751 | 6.62-7.57 | Heat (Ambient temperature from weather monitoring stations) | At delivery | Birth region, obstetric complications, meconium in the amniotic fluid, premature rupture of membrane, placental abruption, placenta previa, massive bleeding, seizure at delivery, precipitating delivery, breech presentation/mal-presentation, cord prolapse, prolonged labor |
| 4. | Wu 2023 | Effects of ambient temperature and relative humidity on preterm birth during early pregnancy and before parturition in China from 2010 to 2018: a population-based large-sample cohort study | China | January 2010–December 2018 | Cohort study | 205771 | 8.75 | Heat/Heat Stress (Meterological data from China National Meteorological Information Center) | 1 week of pregnancy, 4 weeks of pregnancy, 4 weeks before delivery, and 1 week before delivery | Covariates related to preterm birth, namely age, education, history of delivery, history of adverse pregnancy outcomes, BMI, anemia, habit of smoking during pregnancy, habit of drinking alcohol during pregnancy, and fetal sex |
| 5. | Wang 2023 | Effects of gestational ambient extreme temperature exposures on the risk of preterm birth in China: A sibling-matched study based on a multi-center prospective cohort | China | March 2013– December 2018 | Cohort study | 10826 | 3.71 | Heat (Meteorological data across all sites to a fine-scale product at a 1 × 1 km spatial resolution obtained from China Meteorological Data Service Center) | Three trimesters, the entire pregnancy, one week and four weeks before delivery | Maternal information, the residential address at the sub-district level, demographic characteristics such as maternal age, education, pre-pregnancy height and weight, lifestyle characteristics like drinking and smoking, parity, prenatal care visits, and infants' information including birth date, infant sex, birth weight, delivery time, delivery mode |
| 6. | Shankar 2023 | Associations between ambient temperature and pregnancy outcomes from three south Asian sites of the Global Network Maternal Newborn Health Registry: A retrospective cohort study | India, Pakistan | 2014–2020 | Cohort study | 126273 | 16.4 | Heat (Daily maximum air temperatures were obtained from automated observation systems) | At enrolment during pregnancy, within 72 hours of delivery and at 42 days postpartum | Not mentioned |
| 7. | Qian 2016 | Ambient air pollution and preterm birth: A prospective birth cohort study in Wuhan, China | China | June 10, 2011–June 9, 2013 | Cohort study | 95911 | 4.5 | PM 2.5 | 7-14 days pre-conception, each pregnancy month, every trimester, entire pregnancy duration | Maternal age, maternal educational attainment, maternal occupation, parity, gravidity, infant sex, season of conception, and temperature at conception |
| 8. | Li 2018 | Effect of airborne particulate matter of 2.5 µm or less on preterm birth: A national birth cohort study in China | China | December 1st, 2013- November 30th, 2014 | Cohort study | 1240978 | 8.1 | PM 2.5 | Every trimester of pregnancy | Maternal age, registration areas, educational time, employment status, pre-pregnancy body mass index, organic solvent/heavy metals/pesticide exposure, drinking and smoking (active and passive) during pregnancy and conception season, mode of delivery, baby gender |
| 9. | Liu 2019 | The association between air pollution and preterm birth and low birth weight in Guangdong, China | China | January 1, 2014–December 31, 2015 | Cohort study | 86139 | 0.79 | PM 2.5 | Each trimester of pregnancy | Maternal age, education level, occupation, registered residence, gestational age, infant sex, childbirth time, month of conception and parity |
| 10. | Li 2020 | Urban-rural disparity in the relationship between ambient air pollution and preterm birth | China | 2014 | Cross sectional | 429865 | 2.98 | PM 2.5 | Entire pregnancy duration | Minority status, maternal age, family income, parity, education level, baby gender |
| 11. | Liu 2019 | Effects of prenatal exposure to air particulate matter on the risk of preterm birth and roles of maternal and cord blood LINE-1 methylation: A birth cohort study in Guangzhou, China | China | 2016–2019 | Cohort study | 4101 | 5.7 | PM 2.5 | First trimester onwards till delivery | Maternal socio-demographic characteristics, lifestyle factors, infant sex, pre pregnancy BMI, parity, gravidity, season of conception, gestational hypertension, gestational diabetes, and adverse pregnancy history |
| 12. | Sun 2019 | Maternal ambient air pollution exposure with spatial-temporal variations and preterm birth risk assessment during 2013-2017 in Zhejiang Province, China | China | 2013-2017 | Cross sectional study | 6275 | 5.9 | Heat (Derived from ground monitoring stations), PM 2.5 | Each pregnancy month, trimester, and entire pregnancy duration | Maternal age, height, weight, body mass index (BMI), occupation, gestational hypertension (occur or not), and gender of the neonates |
| 13. | Wang 2020 | Independent and combined effects of heatwaves and PM2:5 on preterm birth in Guangzhou, China: A survival analysiIs | China | January 2015 - July 2017 | Cross Sectional | 215059 | 5.17 | Heat (Daily meteorological data from local monitoring stations from Guangdong Meteorological Service Center), PM 2.5 | Final Gestational week | Maternal age, parity, and month of birth, relative humidity exposure |
| 14. | Yuan 2020 | Critical windows for maternal fine particulate matter exposure and adverse birth outcomes: The Shanghai birth cohort study | China | 2013-2016 | Cohort study | 3692 | 4.6 | PM 2.5 | Each trimester of pregnancy | Maternal age, pre-pregnancy BMI, maternal education level, parity, infant sex, passive smoking status, drinking status, individual mean temperature and humidity during the week of conception, birth season, fuel types for cooking and kitchen exhaust equipment usage |
| 15. | Chen 2021 | The association between preterm birth and ambient air pollution exposure in Shiyan, China, 2015-2017 | China | 2015-2017 | Cohort study | 13111 | 4.68 | PM 2.5 | Every trimester of pregnancy | Maternal age, maternal education, gravidity, parity, and fetal gender. |
| 16. | Wang 2020 | Ambient temperature and the risk of preterm birth: A national birth cohort study in the mainland China | China | 2013-2014 | Cohort study | 1281859 | 8.1 | Heat (Daily mean temperature data from weather stations of China Meteorological Data Sharing Service System) | Every trimester of pregnancy | Maternal age, urban residence, education, occupation, body mass index before conception, lifestyle, organic solvent/ heavy metal/pesticide exposure during pregnancy and season of conception |
| 17. | Tapia 2020 | Association between maternal exposure to particulate matter (PM2.5) and adverse pregnancy outcomes in Lima, Peru | Peru | 2012-2016 | Cross sectional study | 123034 | 6.5 | PM 2.5 | Average exposure during each trimester and the entire pregnancy | Maternal age, pre-conception BMI, pre-eclampsia (Yes/No), number of antenatal visits, lifestyle, gestational diabetes (Yes/No) |
| 18. | Li 2021 | Long-term impact of ambient air pollution on preterm birth in Xuzhou, China: a time series study | China | January 1, 2016 - June 30, 2019 | Cross-sectional study | 120446 | 4.48 | PM 2.5 | Average weekly exposure per trimester of pregnancy | Maternal age and season of delivery |
| 19. | Zhou 2022 | Association between Maternal Exposure to Ambient Air Pollution and the Risk of Preterm Birth: A Birth Cohort Study in Chongqing, China, 2015-2020 | China | 2015-2020 | Cohort study | 572116 | 5.88 | PM 2.5 | Mean concentrations during the entire pregnancy and every trimester | Mean temperature, humidity, maternal and paternal age, birth weight |
| 20. | Zhou 2022 | Prenatal exposure to air pollution and the risk of preterm birth in rural population of Henan Province | China | 2014-2016 | Cross sectional study | 679316 | 2.2 | PM 2.5 | Mean concentrations during the entire pregnancy and every trimester | Maternal age, pregnancy BMI, education, lifestyle, parity, season of conception, sex of the neonates, ambient temperature and relative humidity |
| 21. | Fang 2022 | Maternal exposures to fine and ultrafine particles and the risk of preterm birth from a retrospective study in Beijing, China | China | 2014-2017 | Cross-sectional study | 24001 | 4.4 | PM 2.5, Black Carbon | The entire duration of the pregnancy | Maternal age, maternal ethnicity, gravidity, parity, fetal maternal weight at the 12^th^ gestational week, gestational weight gain, year and season of conception |
| 22. | He 2022 | Composition of fine particulate matter and risk of preterm birth: A nationwide birth cohort study in 336 Chinese cities | China | January 2010 - December 2015 | Cohort study | 3723139 | 7.7 | PM 2.5, Black Carbon | Each trimester, and entire pregnancy duration | Maternal age, educational attainment, second-hand smoke exposure during pregnancy, parity, pre-pregnancy BMI, infant’s sex, and season of conception. |
| 23. | Wang 2022 | Assessment of the association between prenatal exposure to multiple ambient pollutants and preterm birth: A prospective cohort study in Jinan, east China | China | June 2018 - November 2019 | Cohort study | 424 | 4.00 | PM 2.5 | Entire duration of pregnancy | Maternal age, BMI, education background, menstruation status, age at menarche, gestation, parity, paternal cigarette and alcohol consumption, and sex of the newborn. |
| 24. | He 2022 | Relationship between ambient air pollution and preterm birth: a retrospective birth cohort study in Yan'an, China | China | January 2018 -December 2019 | Cohort study | 10722 | 4.29 | PM 2.5 | Each trimester, and entire pregnancy duration | Maternal age, parity, gravidity, season of conception, hypertensive disorders of pregnancy, menstrual cycle, Apgar score Pregnancy complications |
| 25. | Wang 2023 | The relationship between air pollutants and preterm birth in a typical river valley city: A retrospective cohort study | China | January, 2018 – December 2019 | Cohort study | 6133 | 6.9 | PM 2.5 | Each trimester, and entire pregnancy duration | Maternal age, gravidity, parity, occupation, number of cesarean sections, season of last menstruation, menstrual cycle, complications, comorbid diseases, and hypertensive disorders in pregnancy |
| 26. | Ming 2023 | The short-term effects of air pollution exposure on preterm births in Chongqing, China: 2015-2020 | China | 2015–2020 | Cross-Sectional study | 598018 | 5.86 | PM 2.5 | Entire duration of pregnancy | Mean temperature and relative humidity. |
| 27. | Qiu 2023 | Third trimester as the susceptibility window for maternal PM2.5 exposure and preterm birth: A nationwide surveillance-based association study in China | China | 2013-2019 | Cohort study | 2294188 | 3.61 | PM 2.5 | Each trimester, and entire pregnancy duration | Neonate sex, maternal ethnicity, maternal age, parity, month and year of conception, residential status, residential area, economic development level, temperature and relative humidity |
| 28. | Mitku 2023 | Impact of ambient air pollution exposure during pregnancy on adverse birth outcomes: generalized structural equation modeling approach | South Africa | 2013–2017 | Cohort study | 656 | 17.22 | PM 2.5 | Entire pregnancy duration. | Gestational weight gain, BMI in the first trimester, and HIV status, maternal age, infant gender, socio-economic status, lifestyle, physical activity |
| 29. | Yu 2024 | Association of residential greenness, air pollution with adverse birth outcomes: Results from 61,762 mother-neonatal pairs in project ELEFANT (2011-2021) | China | 2011-2021 | Cohort study | 61762 | 3.19 | PM 2.5 | Each trimester, and entire pregnancy duration, and 180 days prior to conception | Maternal age, gravidity, parity, maternal residence, conception season, overweight and self-reported stress |
| 30. | Zhang 2024 | Independent and interaction effects of prenatal exposure to high AQI and extreme Humidex on the risk of preterm birth: A large sample population study in northern China | China | 2019-2020 | Cross sectional study | 34365 | 5.74 | Heat (Daily temperature and humidity data sourced from the Shandong Environmental Information Monitoring Center and the China Meteorological Science Data Sharing Service), PM 2.5 | Each trimester, and entire pregnancy duration | Maternal age, marital status, ethnicity, occupation status gravidity, parity, number of abortions, season of conception, pre-pregnancy BMI |
| 31. | Bachwenkizi 2022 | Maternal exposure to fine particulate matter and preterm birth and low birth weight in Africa | 15 African countries (Tanzania, Cameroon, Zimbabwe, Mali, Nigeria, Chad, Benin, South Africa, Burundi, Uganda, Ethiopia, Guinea, Zambia, Angola, and Malawi) | 2005–2015 | Cohort study | 131594 | 3.3 | PM 2.5 | Entire duration of pregnancy | Maternal age, educational status, lifestyle, number of antenatal visits, parity, infant gender, cooking energy source, socio-economic status, residential area |
| 32. | He 2016 | Ambient temperature and the risk of preterm birth in Guangzhou, China (2001-2011) | China | 2001–2011 | Cross-sectional study | 838146 | 5.6 | Heat (Daily temperature and humidity data sourced from Guangzhou Meteorological Bureau) | The last week of pregnancy, the last 4 weeks of pregnancy, late pregnancy (gestational week 20 onward), and the entire pregnancy | Maternal age, maternal education level, parity, baby’s sex, month and year of conception |

##

**Supplementary Table 4.** Results of the Sensitivity Analysis for Heat Studies - Leave-one-out analysis

| **Exposure Window** | **Study Removed** | **Estimate** | **SE** | **Z-value** | **P-value** | **CI Lower** | **CI Upper** | **Q** | **Qp** | **Tau²** | **I² (%)** | **H²** |
| --- | --- | --- | --- | --- | --- | --- | --- | --- | --- | --- | --- | --- |
| **T1 (Random Effects)** | Wang 2024 | 0.2751 | 0.103 | 2.6725 | 0.0075 | 0.0734 | 0.4769 | 441.3507 | 0.0 | 0.0584 | 98.87 | 88.27 |
| **T1 (Random Effects)** | Zheng 2018 | 0.2639 | 0.1041 | 2.5354 | 0.0112 | 0.0599 | 0.4678 | 439.4017 | 0.0 | 0.0595 | 98.86 | 87.88 |
| **T1 (Random Effects)** | Weng 2018 | 0.2794 | 0.125 | 2.236 | 0.0253 | 0.0345 | 0.5244 | 441.0035 | 0.0 | 0.0873 | 98.87 | 88.2 |
| **T1 (Random Effects)** | Wu 2023 | 0.2509 | 0.109 | 2.3016 | 0.0214 | 0.0372 | 0.4646 | 412.1436 | 0.0 | 0.0654 | 98.79 | 82.43 |
| **T1 (Random Effects)** | Wang 2023 | 0.2424 | 0.0972 | 2.4943 | 0.0126 | 0.0519 | 0.433 | 439.9431 | 0.0 | 0.0554 | 98.86 | 87.99 |
| **T1 (Random Effects)** | Shankar 2023 | 0.2983 | 0.1028 | 2.901 | 0.0037 | 0.0968 | 0.4999 | 235.5408 | 0.0 | 0.0576 | 97.88 | 47.11 |
| **T1 (Random Effects)** | Wang 2020 | 0.166 | 0.0533 | 3.1124 | 0.0019 | 0.0615 | 0.2705 | 93.3178 | 0.0 | 0.0137 | 94.64 | 18.66 |
| **T2 (Random Effects)** | Wang 2024 | 0.0918 | 0.0867 | 1.058 | 0.2901 | -0.0782 | 0.2618 | 80.7999 | 0.0 | 0.025 | 96.29 | 26.93 |
| **T2 (Random Effects)** | Zheng 2018 | 0.1691 | 0.0765 | 2.2097 | 0.0271 | 0.0191 | 0.319 | 47.7984 | 0.0 | 0.0182 | 93.72 | 15.93 |
| **T2 (Random Effects)** | Wang 2023 | 0.0724 | 0.0767 | 0.9435 | 0.3454 | -0.078 | 0.2228 | 79.6479 | 0.0 | 0.022 | 96.23 | 26.55 |
| **T2 (Random Effects)** | Shankar 2023 | 0.1284 | 0.1282 | 1.0013 | 0.3167 | -0.1229 | 0.3796 | 74.7236 | 0.0 | 0.0578 | 95.99 | 24.91 |
| **T2 (Random Effects)** | Wang 2020 | 0.0405 | 0.0717 | 0.5646 | 0.5724 | -0.1001 | 0.1811 | 29.8533 | 0.0 | 0.0155 | 89.95 | 9.95 |
| **T3 (Random Effects)** | Zheng 2018 | 0.3715 | 0.1375 | 2.7025 | 0.0069 | 0.1021 | 0.641 | 245.8729 | 0.0 | 0.0691 | 98.78 | 81.96 |
| **T3 (Random Effects)** | Wu 2023 | 0.2241 | 0.078 | 2.874 | 0.0041 | 0.0713 | 0.3769 | 45.1101 | 0.0 | 0.0194 | 93.35 | 15.04 |
| **T3 (Random Effects)** | Wang 2023 | 0.2036 | 0.1163 | 1.7503 | 0.0801 | -0.0244 | 0.4316 | 218.3475 | 0.0 | 0.0529 | 98.63 | 72.78 |
| **T3 (Random Effects)** | Wang 2020 | 0.4153 | 0.1348 | 3.0821 | 0.0021 | 0.1512 | 0.6794 | 98.26 | 0.0 | 0.0655 | 96.95 | 32.75 |
| **T3 (Random Effects)** | He 2016 | 0.4101 | 0.1536 | 2.6698 | 0.0076 | 0.109 | 0.7112 | 250.2333 | 0.0 | 0.0872 | 98.8 | 83.41 |
| **EP (Fixed Effects)** | Zheng 2018 | 1.5195 | 0.1551 | 9.7965 | 0.0 | 1.2155 | 1.8235 | 0.0 | 1.0 | 0.0 | 0.0 | 1.0 |
| **EP (Fixed Effects)** | Wang 2023 | 0.9439 | 0.1326 | 7.1173 | 0.0 | 0.684 | 1.2038 | 0.0 | 1.0 | 0.0 | 0.0 | 1.0 |

**Supplementary Table 5.** Results of the Sensitivity Analysis for PM2.5 Studies - Leave-one-out analysis

| **Exposure Window** | **Study Removed** | **Estimate** | **SE** | **Z-value** | **P-value** |
| --- | --- | --- | --- | --- | --- |
| **T1 (Random Effects)** | Li 2018 | 0.0146 | 0.0095 | 1.5368 | 0.1243 |
| **T1 (Random Effects)** | Liu 2019 | 0.0188 | 0.0202 | 0.9301 | 0.3523 |
| **T1 (Random Effects)** | Yuan 2020 | 0.0254 | 0.0188 | 1.3523 | 0.1763 |
| **T1 (Random Effects)** | Chen 2021 | 0.0235 | 0.0199 | 1.1806 | 0.2377 |
| **T1 (Random Effects)** | Tapia 2020 | 0.023 | 0.0195 | 1.1792 | 0.2383 |
| **T1 (Random Effects)** | Zhou 2022.1 | 0.0286 | 0.0186 | 1.5371 | 0.1243 |
| **T1 (Random Effects)** | Zhou 2022.2 | 0.0181 | 0.0204 | 0.888 | 0.3745 |
| **T1 (Random Effects)** | He 2022 | 0.0261 | 0.0194 | 1.3454 | 0.1785 |
| **T2 (Random Effects)** | Li 2018 | 0.0262 | 0.0115 | 2.2662 | 0.0234 |
| **T2 (Random Effects)** | Liu 2019.1 | 0.0332 | 0.016 | 2.0697 | 0.0385 |
| **T2 (Random Effects)** | Liu 2019.2 | 0.0296 | 0.0157 | 1.8846 | 0.0595 |
| **T2 (Random Effects)** | Yuan 2020 | 0.0363 | 0.0156 | 2.3321 | 0.0197 |
| **T2 (Random Effects)** | Chen 2021 | 0.0331 | 0.0164 | 2.0193 | 0.0435 |
| **T2 (Random Effects)** | Tapia 2020 | 0.0386 | 0.0164 | 2.3587 | 0.0183 |
| **T2 (Random Effects)** | Zhou 2022.1 | 0.0388 | 0.0165 | 2.3529 | 0.0186 |
| **T2 (Random Effects)** | Zhou 2022.2 | 0.0206 | 0.0158 | 1.3014 | 0.1931 |
| **T3 (Random Effects)** | Li 2018 | 0.0716 | 0.0225 | 3.1795 | 0.0015 |
| **T3 (Random Effects)** | Liu 2019 | 0.0572 | 0.0161 | 3.5511 | 0.0004 |
| **T3 (Random Effects)** | Yuan 2020 | 0.0546 | 0.0154 | 3.5327 | 0.0004 |
| **T3 (Random Effects)** | Chen 2021 | 0.054 | 0.0162 | 3.3247 | 0.0009 |
| **T3 (Random Effects)** | Tapia 2020 | 0.064 | 0.0162 | 3.9488 | 0.0001 |
| **T3 (Random Effects)** | Zhou 2022.1 | 0.0624 | 0.0168 | 3.7077 | 0.0002 |
| **EP (Random Effects)** | Qian 2016 | 0.0882 | 0.0173 | 5.0903 | 0.0 |
| **EP (Random Effects)** | Li 2018 | 0.0864 | 0.0173 | 4.9794 | 0.0 |
| **EP (Random Effects)** | Liu 2019.1 | 0.0914 | 0.0173 | 5.2744 | 0.0 |
| **EP (Random Effects)** | Li 2020 | 0.0934 | 0.0185 | 5.0576 | 0.0 |
| **EP (Random Effects)** | Sun 2019 | 0.0848 | 0.0171 | 4.9708 | 0.0 |
| **EP (Random Effects)** | Chen 2021 | 0.0788 | 0.017 | 4.6311 | 0.0 |

**Supplementary Figure 1.** Funnel plot for heat studies across all trimesters.
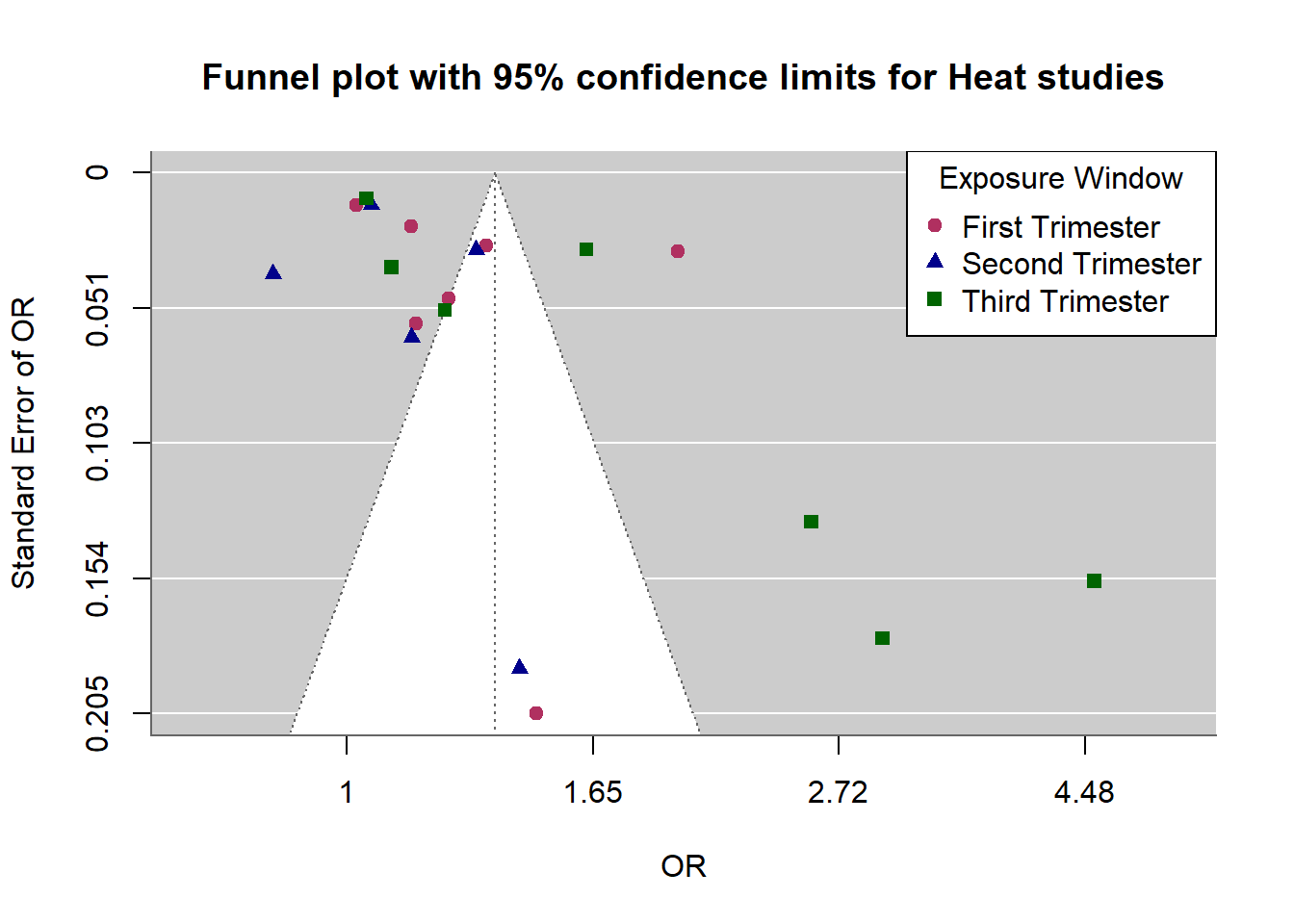


**Supplementary Figure 2.** Funnel plot with Trim and Fill Effects for heat studies across all gestational periods.


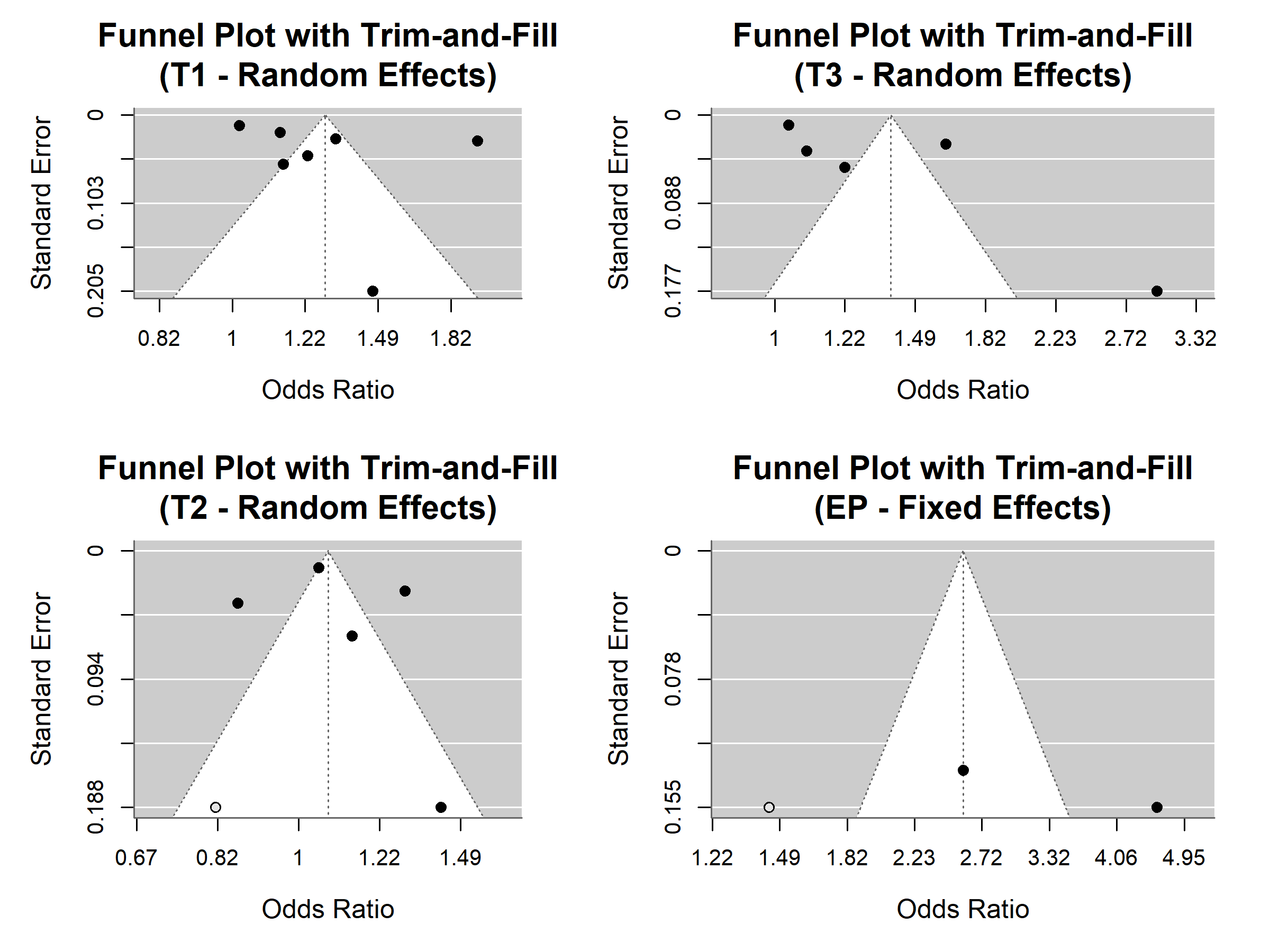


**Supplementary Figure 3.** Funnel plot with Trim and Fill Effects for heat studies across all trimesters.


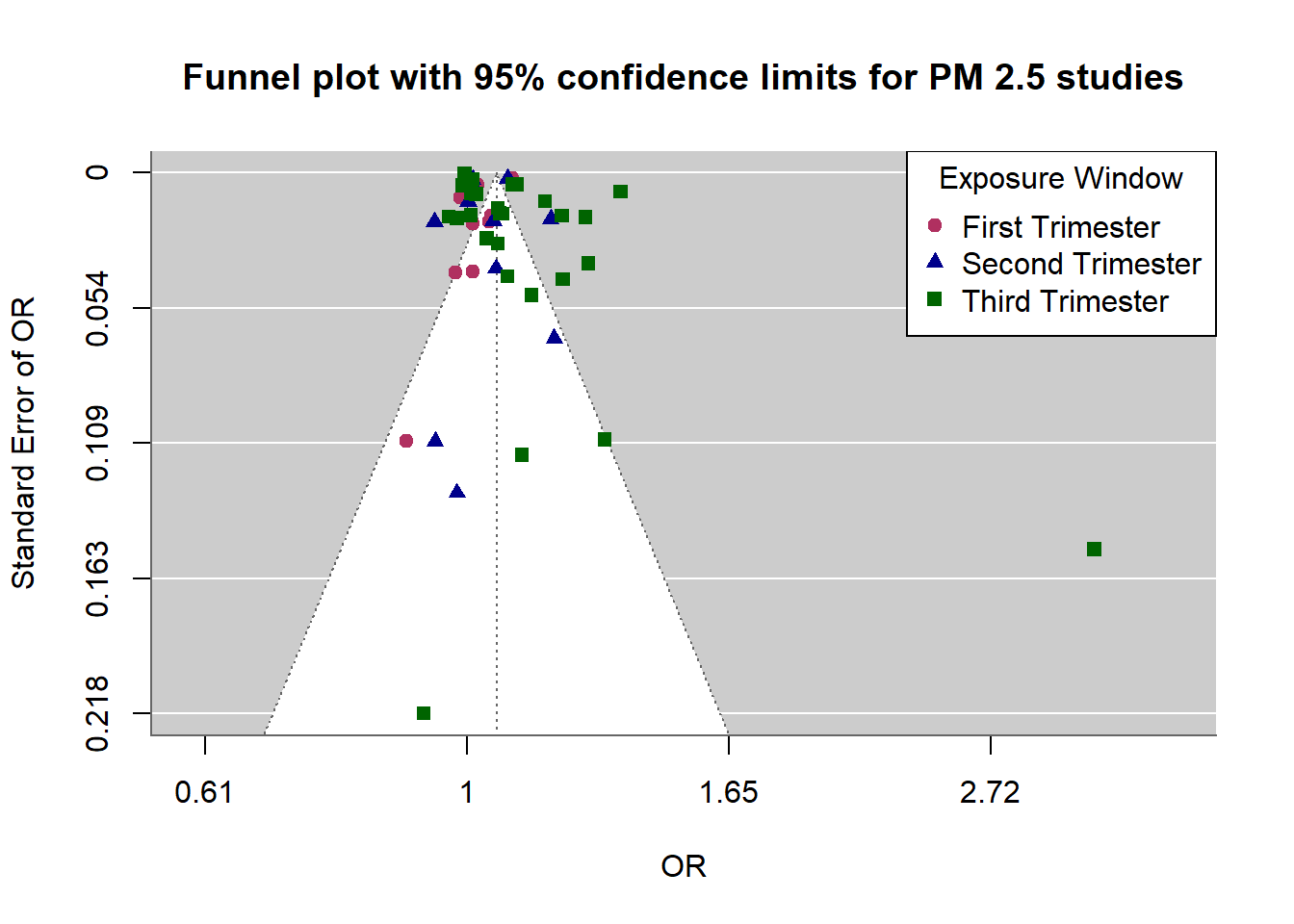


**Supplementary Figure 4.** Funnel plot for PM2.5 studies across all gestational periods.


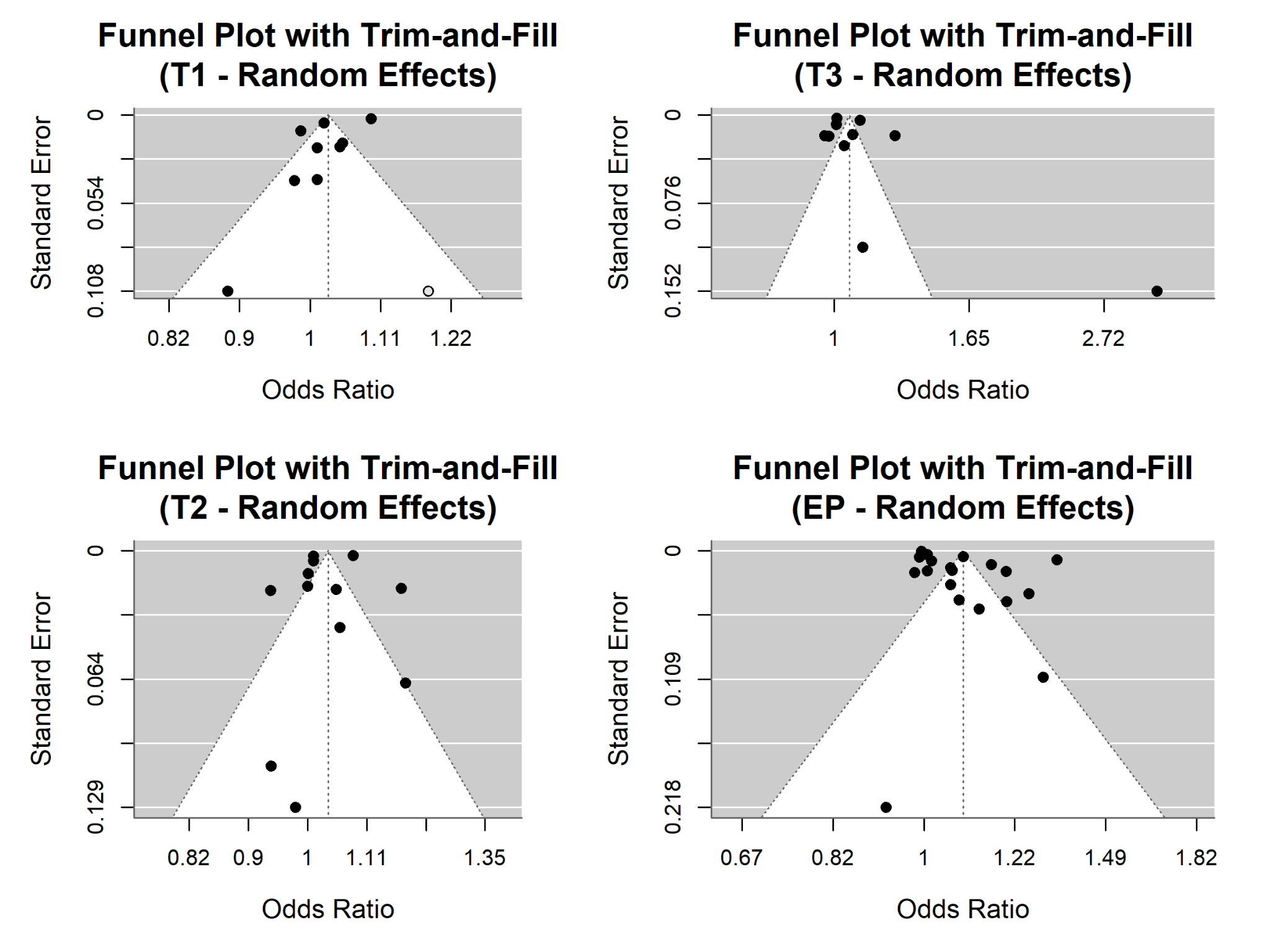

Supplement: online supplemental file 1 [file bmjpo-9-1-s001.docx]
