# Supplementary material for: Association between heat and air pollution (PM2.5 and black carbon) exposure in pregnancy and preterm birth in low- and middle-income countries: a systematic review and meta-analysis
Source: BMJ Paediatr Open. 2025 Sep 14;9(1):e003428. doi: 10.1136/bmjpo-2025-003428 (PMC12434743; doi:10.1136/bmjpo-2025-003428)
Supplement: Supplementary file 2 [file bmjpo-2025-003428.reviewer_comments.pdf]

PEER REVIEW HISTORY

BMJ Paediatrics Open publishes all reviews undertaken for accepted manuscripts. Reviewers are asked to complete a checklist review form and are provided with free text boxes to elaborate on their assessment. These free text comments are reproduced below.

ARTICLE DETAILS

**TITLE (PROVISIONAL)**	Association Between Heat and Air Pollution (PM2.5 and Black Carbon) Exposure in Pregnancy and Preterm Birth in Low and Middle-Income Countries: A Systematic Review & Meta-Analysis
**AUTHORS**	Raghavan, Sreevatsan; Isaac, Tanya; Arya, Divya; Flores, Gabriela Cipriano; Shanmugam, Rekha; Na, Ayushi; Desiraju, Bapu Koundinya; Venugopal, Vidhya; Thiruvengadam, Ramachandran; Kshetrapal, Pallavi; Wadhwa, Nitya; Downey, Laura; Hirst, Jane; Bhatnagar, Shinjini; (HiP) - India Group, Heat in Pregnancy

VERSION 1 - REVIEW

**REVIEWER NAME**	David Warburton
**REVIEWER AFFILIATION**	None disclosed
**REVIEWER CONFLICT OF INTEREST**	N/A
**AI DISCLOSURE**	No
**DATE REVIEW RETURNED**	30-Mar-2025

**GENERAL COMMENTS**	This is a useful<br>Meta analysis of the published evidence on climate heat and black carbon effects on preterm birth.<br>I have no revisions,
